# Supplementary material for: Cryopreservation of cerebrospinal fluid cells preserves the transcriptional landscape for single-cell analysis
Source: J Neuroinflammation. 2024 Mar 23;21:71. doi: 10.1186/s12974-024-03047-1 (PMC10960996; doi:10.1186/s12974-024-03047-1)
Supplement: Supplementary file 1 — Additional file 1: Fig. S1. Representative images of the CSF cell pellet visible post-centrifugation. See image file. A sample devoid of RBC contamination is seen on the left, and one with substantial RBC contamination is seen on the right. Table S1. Donor demographics and sample information. See Excel file. Subject IDs, Age, Sex, and number of individual reactions tested from FRE, FBS, REC, and DNA samples listed. Table S2. Quality control metrics pre- and post-filtration. See Excel file. QC filtering: Cells having 200 – 4500 umi counts, < 20% mito genes detected were retained. 2000 high variable genes were used for clustering. Columns: Total_cells.before.filtering: cells pre-filtering; cells after filtering: cells remaining after application of above filters; Total_genes: Genes per sample, avg_numi: average number of umi counts before filtering; numi after filtering: average number of umi counts after filtering; avg_mito: average mitochondrial genes in each sample before filtering; mito after filtering: average number of mitochondrial genes in each sample after filtering. Fig. S2. Cellular features, UMI, mitochondrial reads, and cellular stress-related gene expression. See image file. All graphs include data from CSF A, B, and C. A–C Density plot showing the distribution of the number of transcriptional features (genes), number of Unique Molecular Identifiers (UMI), and percentage of overall gene expression attributed to mitochondrial genes, respectively. D–F Violin plots of HSPA1A, HSPA1B, and HSP90AA1 normalized gene expression levels, respectively. Table S3. Cell type designations, proportions and mean predicted scores. See Excel file. Mean predicted score is the Azimuth-derived confidence score for a given annotation. ‘All’ refers to the total of all cell types (L1 or L2). Columns E-L reference L1 designations (used throughout the manuscript), and columns M-AP list L2 designations (not used elsewhere in the manuscript). Summary information for samples by fresh [file 12974_2024_3047_MOESM1_ESM.zip › Supplementary_Information_Kodali_2024_ESM.docx]

**SUPPLEMENTARY INFORMATION**

**S1 Fig. Representative images of the CSF cell pellet visible post-centrifugation.** See image file. A sample devoid of RBC contamination is seen on the left, and one with substantial RBC contamination is seen on the right.

**S1 Table. Donor demographics and sample information.** See Excel file. Subject IDs, Age, Sex, and number of individual reactions tested from FRE, FBS, REC, and DNA samples listed.

**S2 Table. Quality control metrics pre- and post-filtration.** See Excel file. QC filtering: Cells having 200 – 4500 umi counts, < 20% mito genes detected were retained. 2000 high variable genes were used for clustering. Columns: Total_cells.before.filtering: cells pre-filtering; cells after filtering: cells remaining after application of above filters; Total_genes: Genes per sample, avg_numi: average number of umi counts before filtering; numi after filtering: average number of umi counts after filtering; avg_mito: average mitochondrial genes in each sample before filtering; mito after filtering: average number of mitochondrial genes in each sample after filtering.

**S2 Fig. Cellular features, UMI, mitochondrial reads, and cellular stress-related gene expression.** See image file. All graphs include data from CSF A, B, and C. (A-C) Density plot showing the distribution of the number of transcriptional features (genes), number of Unique Molecular Identifiers (UMI), and percentage of overall gene expression attributed to mitochondrial genes, respectively. (D-F) Violin plots of HSPA1A, HSPA1B, and HSP90AA1 normalized gene expression levels, respectively.

**S3 Table.** **Cell type designations, proportions and mean predicted scores.** See Excel file. Mean predicted score is the Azimuth-derived confidence score for a given annotation. ‘All’ refers to the total of all cell types (L1 or L2). Columns E-L reference L1 designations (used throughout the manuscript), and columns M-AP list L2 designations (not used elsewhere in the manuscript). Summary information for samples by fresh or cryopreservation method can be found in the lowest rows.

**S3 Fig.** **Concordance of cell type markers between cryopreserved and freshly run samples**. See image file. (A) UMAP of all CSF cells labeled by degree of expression of a combination of CX3CR1, CSF1R, SLC2A5, MARCKS, and P2RY13, which has been shown to relate to the microglial homeostatic gene signature. (B) Dot plot comparison of cell-type specific enrichment of marker gene expression in FRE, FBS, and REC protocols for samples CSF A, B, and C. Cell-type specific marker gene expression and associated cell types are listed on the y-axis, and Azimuth reference atlas cell designations at L1 resolution are listed on the x-axis.

**S4 Table.** **Dendrogram adjacency matrix of gene expression by cell type.** See Excel file. See individual sheets for overall and cell-specific matrices. The order of the donors and fresh/cryopreservation condition is based on the unweighted Euclidian distance between samples as visualized on the dendrograms (Figs 3a and S6).

**S4 Fig.** **FBS versus Recovery comparisons.** See image file. **(**A) UMAP plot of all the cells in samples CSF A, B, C, D, E, F, and G from FBS and REC protocols were annotated to show the cell types that were clustered using Azimuth PBMC reference atlas at L1 resolution. (B) Density plot showing the similar distribution of the number of genes, number of unique transcripts, and percentage of mitochondrial genes in all the cells from FBS and REC protocols in samples CSF A, B C, D, E, F, and G. (C) Stacked bar plot depicting the individual cell type proportions obtained after clustering using Azimuth reference atlas at L1 resolution in samples CSF A, B C, D, E, F and G from FBS and REC protocols.

**S5 Fig. FBS versus FBS with Dnase (DNA) comparisons.** See image file. (A) UMAP plot of all the cells in samples CSF A, C, D, and G from FBS and DNA protocols were annotated to show the cell types clustered using Azimuth PBMC reference atlas at L1 resolution. (B) Density plot showing the similar distribution of the number of genes, number of unique transcripts, and percentage of mitochondrial genes in all the cells from FBS and DNA protocols in samples CSF A, B C, D, E, F, and G. (C) Stacked bar plot depicting the individual cell type proportions obtained after clustering using Azimuth reference atlas at L1 resolution in samples CSF A, C, D, and G from FBS and DNA protocols.

**S6 Fig. Dendrograms of pseudobulk gene expression by cell type and cryopreservation condition.** See image file. Dendrograms showing the hierarchical clustering of relative sample to sample distances in samples CSF A, B, and C in FRE, FBS, and REC protocols by cell type. Note the companion adjacency matrices found in S4 Table.

**S7 Fig. Scatter plots with correlation statistics of FRE vs FBS and FRE vs REC global gene expression in samples CSF A, B and C in CD4+ T-cells.** See image file. Each dot represents a single gene.

**S8 Fig.** **Scatter plots with correlation statistics of FRE vs FBS and FRE vs REC global gene expression in samples CSF A, B and C in CD8+ T-cells.** See image file. Each dot represents a single gene.

**S9 Fig.** **Scatter plots with correlation statistics of FRE vs FBS and FRE vs REC global gene expression in samples CSF A, B and C in B cells.** See image file. Each dot represents a single gene.

**S10 Fig.** **Scatter plots with correlation statistics of FRE vs FBS and FRE vs REC global gene expression in samples CSF A, B and C in monocytes.** See image file. Each dot represents a single gene.

**S11 Fig.** **Scatter plots with correlation statistics of FRE vs FBS and FRE vs REC global gene expression in samples CSF A, B and C in dendritic cells.** See image file. Each dot represents a single gene.

**S12 Fig. Scatter plots with correlation statistics of FRE vs FBS and FRE vs REC global gene expression in samples CSF A, B and C in NK cells.** See image file. Each dot represents a single gene.
