# Supplementary figures and images for: Cryopreservation of cerebrospinal fluid cells preserves the transcriptional landscape for single-cell analysis
Source: J Neuroinflammation. 2024 Mar 23;21:71. doi: 10.1186/s12974-024-03047-1 (PMC10960996; doi:10.1186/s12974-024-03047-1)

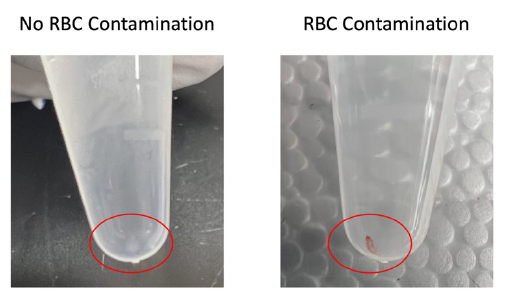

Supplement: Supplementary file 1 — Additional file 1: Fig. S1. Representative images of the CSF cell pellet visible post-centrifugation. See image file. A sample devoid of RBC contamination is seen on the left, and one with substantial RBC contamination is seen on the right. Table S1. Donor demographics and sample information. See Excel file. Subject IDs, Age, Sex, and number of individual reactions tested from FRE, FBS, REC, and DNA samples listed. Table S2. Quality control metrics pre- and post-filtration. See Excel file. QC filtering: Cells having 200 – 4500 umi counts, < 20% mito genes detected were retained. 2000 high variable genes were used for clustering. Columns: Total_cells.before.filtering: cells pre-filtering; cells after filtering: cells remaining after application of above filters; Total_genes: Genes per sample, avg_numi: average number of umi counts before filtering; numi after filtering: average number of umi counts after filtering; avg_mito: average mitochondrial genes in each sample before filtering; mito after filtering: average number of mitochondrial genes in each sample after filtering. Fig. S2. Cellular features, UMI, mitochondrial reads, and cellular stress-related gene expression. See image file. All graphs include data from CSF A, B, and C. A–C Density plot showing the distribution of the number of transcriptional features (genes), number of Unique Molecular Identifiers (UMI), and percentage of overall gene expression attributed to mitochondrial genes, respectively. D–F Violin plots of HSPA1A, HSPA1B, and HSP90AA1 normalized gene expression levels, respectively. Table S3. Cell type designations, proportions and mean predicted scores. See Excel file. Mean predicted score is the Azimuth-derived confidence score for a given annotation. ‘All’ refers to the total of all cell types (L1 or L2). Columns E-L reference L1 designations (used throughout the manuscript), and columns M-AP list L2 designations (not used elsewhere in the manuscript). Summary information for samples by fresh [file 12974_2024_3047_MOESM1_ESM.zip › S1_Fig.tif]

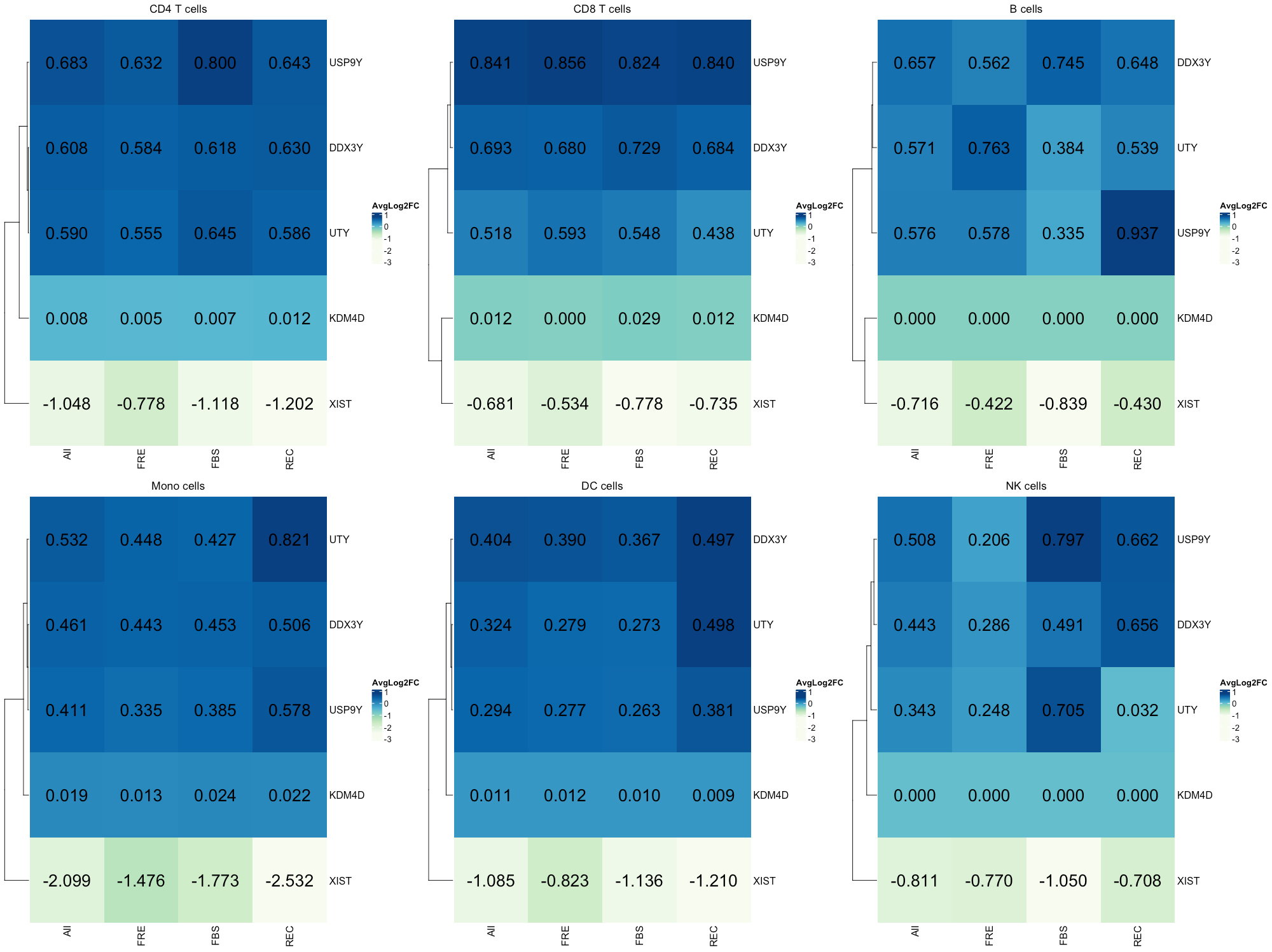

Supplement: Supplementary file 1 — Additional file 1: Fig. S1. Representative images of the CSF cell pellet visible post-centrifugation. See image file. A sample devoid of RBC contamination is seen on the left, and one with substantial RBC contamination is seen on the right. Table S1. Donor demographics and sample information. See Excel file. Subject IDs, Age, Sex, and number of individual reactions tested from FRE, FBS, REC, and DNA samples listed. Table S2. Quality control metrics pre- and post-filtration. See Excel file. QC filtering: Cells having 200 – 4500 umi counts, < 20% mito genes detected were retained. 2000 high variable genes were used for clustering. Columns: Total_cells.before.filtering: cells pre-filtering; cells after filtering: cells remaining after application of above filters; Total_genes: Genes per sample, avg_numi: average number of umi counts before filtering; numi after filtering: average number of umi counts after filtering; avg_mito: average mitochondrial genes in each sample before filtering; mito after filtering: average number of mitochondrial genes in each sample after filtering. Fig. S2. Cellular features, UMI, mitochondrial reads, and cellular stress-related gene expression. See image file. All graphs include data from CSF A, B, and C. A–C Density plot showing the distribution of the number of transcriptional features (genes), number of Unique Molecular Identifiers (UMI), and percentage of overall gene expression attributed to mitochondrial genes, respectively. D–F Violin plots of HSPA1A, HSPA1B, and HSP90AA1 normalized gene expression levels, respectively. Table S3. Cell type designations, proportions and mean predicted scores. See Excel file. Mean predicted score is the Azimuth-derived confidence score for a given annotation. ‘All’ refers to the total of all cell types (L1 or L2). Columns E-L reference L1 designations (used throughout the manuscript), and columns M-AP list L2 designations (not used elsewhere in the manuscript). Summary information for samples by fresh [file 12974_2024_3047_MOESM1_ESM.zip › S13_Fig.jpg]

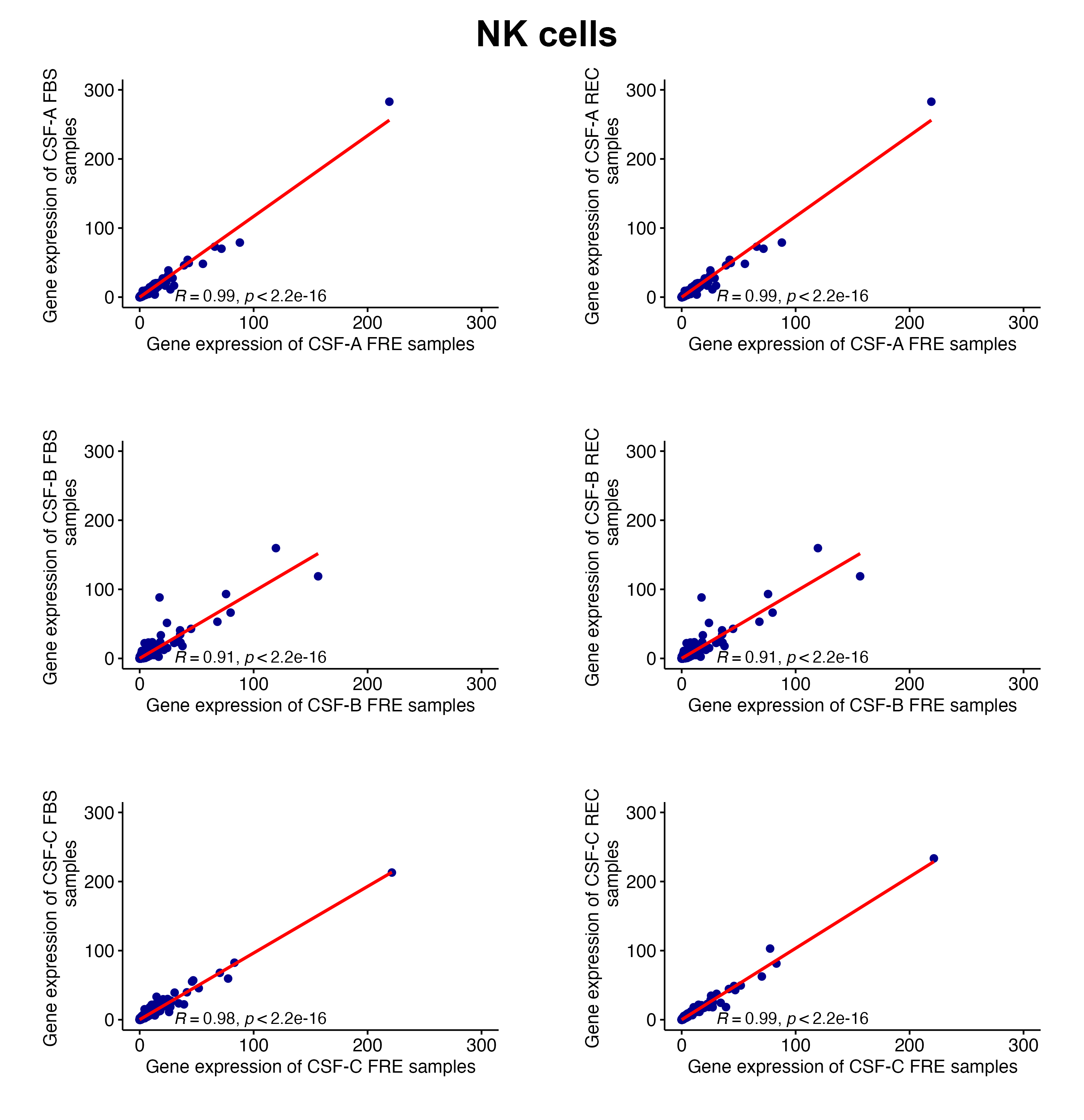

Supplement: Supplementary file 1 — Additional file 1: Fig. S1. Representative images of the CSF cell pellet visible post-centrifugation. See image file. A sample devoid of RBC contamination is seen on the left, and one with substantial RBC contamination is seen on the right. Table S1. Donor demographics and sample information. See Excel file. Subject IDs, Age, Sex, and number of individual reactions tested from FRE, FBS, REC, and DNA samples listed. Table S2. Quality control metrics pre- and post-filtration. See Excel file. QC filtering: Cells having 200 – 4500 umi counts, < 20% mito genes detected were retained. 2000 high variable genes were used for clustering. Columns: Total_cells.before.filtering: cells pre-filtering; cells after filtering: cells remaining after application of above filters; Total_genes: Genes per sample, avg_numi: average number of umi counts before filtering; numi after filtering: average number of umi counts after filtering; avg_mito: average mitochondrial genes in each sample before filtering; mito after filtering: average number of mitochondrial genes in each sample after filtering. Fig. S2. Cellular features, UMI, mitochondrial reads, and cellular stress-related gene expression. See image file. All graphs include data from CSF A, B, and C. A–C Density plot showing the distribution of the number of transcriptional features (genes), number of Unique Molecular Identifiers (UMI), and percentage of overall gene expression attributed to mitochondrial genes, respectively. D–F Violin plots of HSPA1A, HSPA1B, and HSP90AA1 normalized gene expression levels, respectively. Table S3. Cell type designations, proportions and mean predicted scores. See Excel file. Mean predicted score is the Azimuth-derived confidence score for a given annotation. ‘All’ refers to the total of all cell types (L1 or L2). Columns E-L reference L1 designations (used throughout the manuscript), and columns M-AP list L2 designations (not used elsewhere in the manuscript). Summary information for samples by fresh [file 12974_2024_3047_MOESM1_ESM.zip › S12_Fig.jpg]

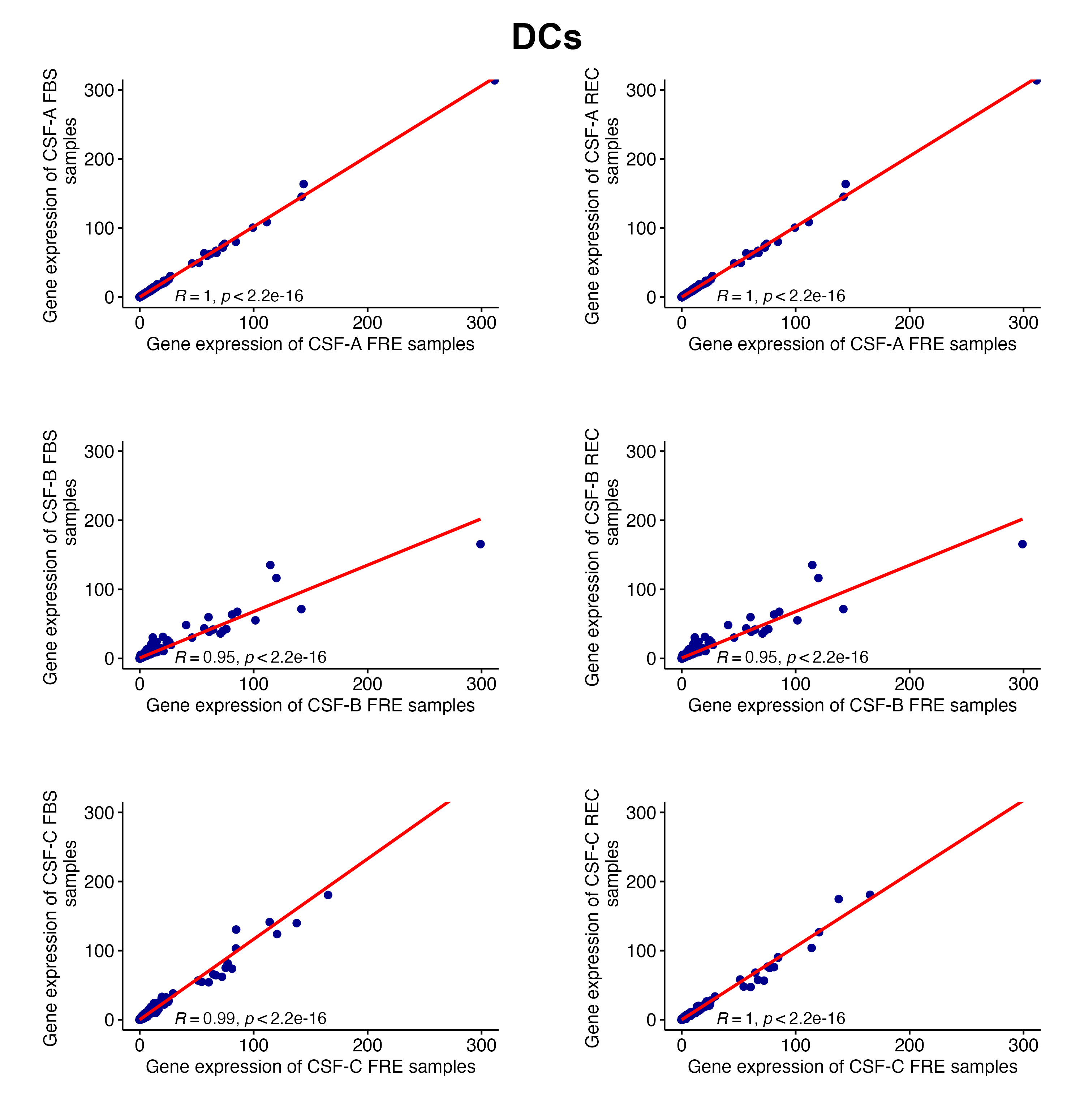

Supplement: Supplementary file 1 — Additional file 1: Fig. S1. Representative images of the CSF cell pellet visible post-centrifugation. See image file. A sample devoid of RBC contamination is seen on the left, and one with substantial RBC contamination is seen on the right. Table S1. Donor demographics and sample information. See Excel file. Subject IDs, Age, Sex, and number of individual reactions tested from FRE, FBS, REC, and DNA samples listed. Table S2. Quality control metrics pre- and post-filtration. See Excel file. QC filtering: Cells having 200 – 4500 umi counts, < 20% mito genes detected were retained. 2000 high variable genes were used for clustering. Columns: Total_cells.before.filtering: cells pre-filtering; cells after filtering: cells remaining after application of above filters; Total_genes: Genes per sample, avg_numi: average number of umi counts before filtering; numi after filtering: average number of umi counts after filtering; avg_mito: average mitochondrial genes in each sample before filtering; mito after filtering: average number of mitochondrial genes in each sample after filtering. Fig. S2. Cellular features, UMI, mitochondrial reads, and cellular stress-related gene expression. See image file. All graphs include data from CSF A, B, and C. A–C Density plot showing the distribution of the number of transcriptional features (genes), number of Unique Molecular Identifiers (UMI), and percentage of overall gene expression attributed to mitochondrial genes, respectively. D–F Violin plots of HSPA1A, HSPA1B, and HSP90AA1 normalized gene expression levels, respectively. Table S3. Cell type designations, proportions and mean predicted scores. See Excel file. Mean predicted score is the Azimuth-derived confidence score for a given annotation. ‘All’ refers to the total of all cell types (L1 or L2). Columns E-L reference L1 designations (used throughout the manuscript), and columns M-AP list L2 designations (not used elsewhere in the manuscript). Summary information for samples by fresh [file 12974_2024_3047_MOESM1_ESM.zip › S11_Fig.jpg]

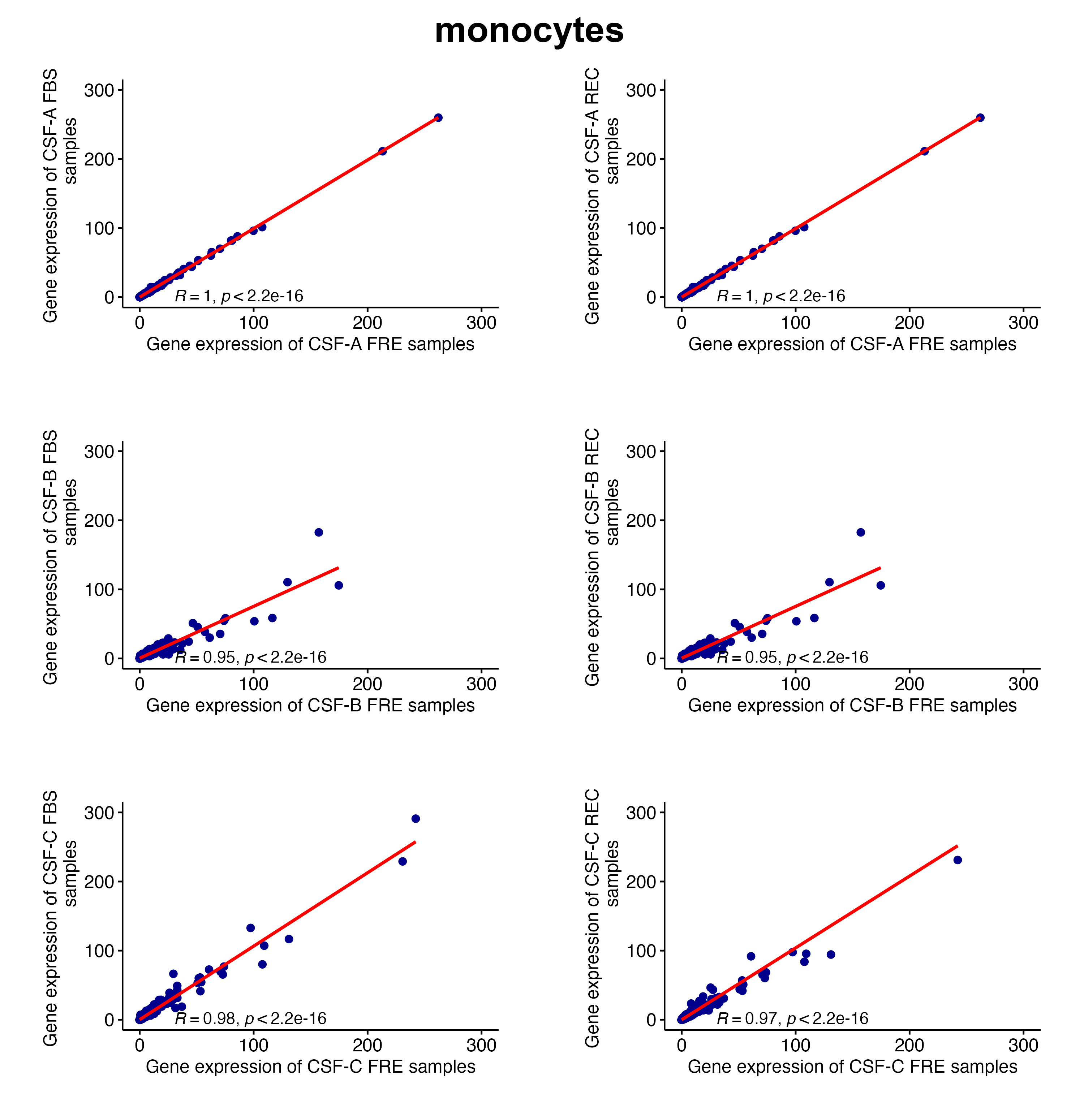

Supplement: Supplementary file 1 — Additional file 1: Fig. S1. Representative images of the CSF cell pellet visible post-centrifugation. See image file. A sample devoid of RBC contamination is seen on the left, and one with substantial RBC contamination is seen on the right. Table S1. Donor demographics and sample information. See Excel file. Subject IDs, Age, Sex, and number of individual reactions tested from FRE, FBS, REC, and DNA samples listed. Table S2. Quality control metrics pre- and post-filtration. See Excel file. QC filtering: Cells having 200 – 4500 umi counts, < 20% mito genes detected were retained. 2000 high variable genes were used for clustering. Columns: Total_cells.before.filtering: cells pre-filtering; cells after filtering: cells remaining after application of above filters; Total_genes: Genes per sample, avg_numi: average number of umi counts before filtering; numi after filtering: average number of umi counts after filtering; avg_mito: average mitochondrial genes in each sample before filtering; mito after filtering: average number of mitochondrial genes in each sample after filtering. Fig. S2. Cellular features, UMI, mitochondrial reads, and cellular stress-related gene expression. See image file. All graphs include data from CSF A, B, and C. A–C Density plot showing the distribution of the number of transcriptional features (genes), number of Unique Molecular Identifiers (UMI), and percentage of overall gene expression attributed to mitochondrial genes, respectively. D–F Violin plots of HSPA1A, HSPA1B, and HSP90AA1 normalized gene expression levels, respectively. Table S3. Cell type designations, proportions and mean predicted scores. See Excel file. Mean predicted score is the Azimuth-derived confidence score for a given annotation. ‘All’ refers to the total of all cell types (L1 or L2). Columns E-L reference L1 designations (used throughout the manuscript), and columns M-AP list L2 designations (not used elsewhere in the manuscript). Summary information for samples by fresh [file 12974_2024_3047_MOESM1_ESM.zip › S10_Fig.jpg]

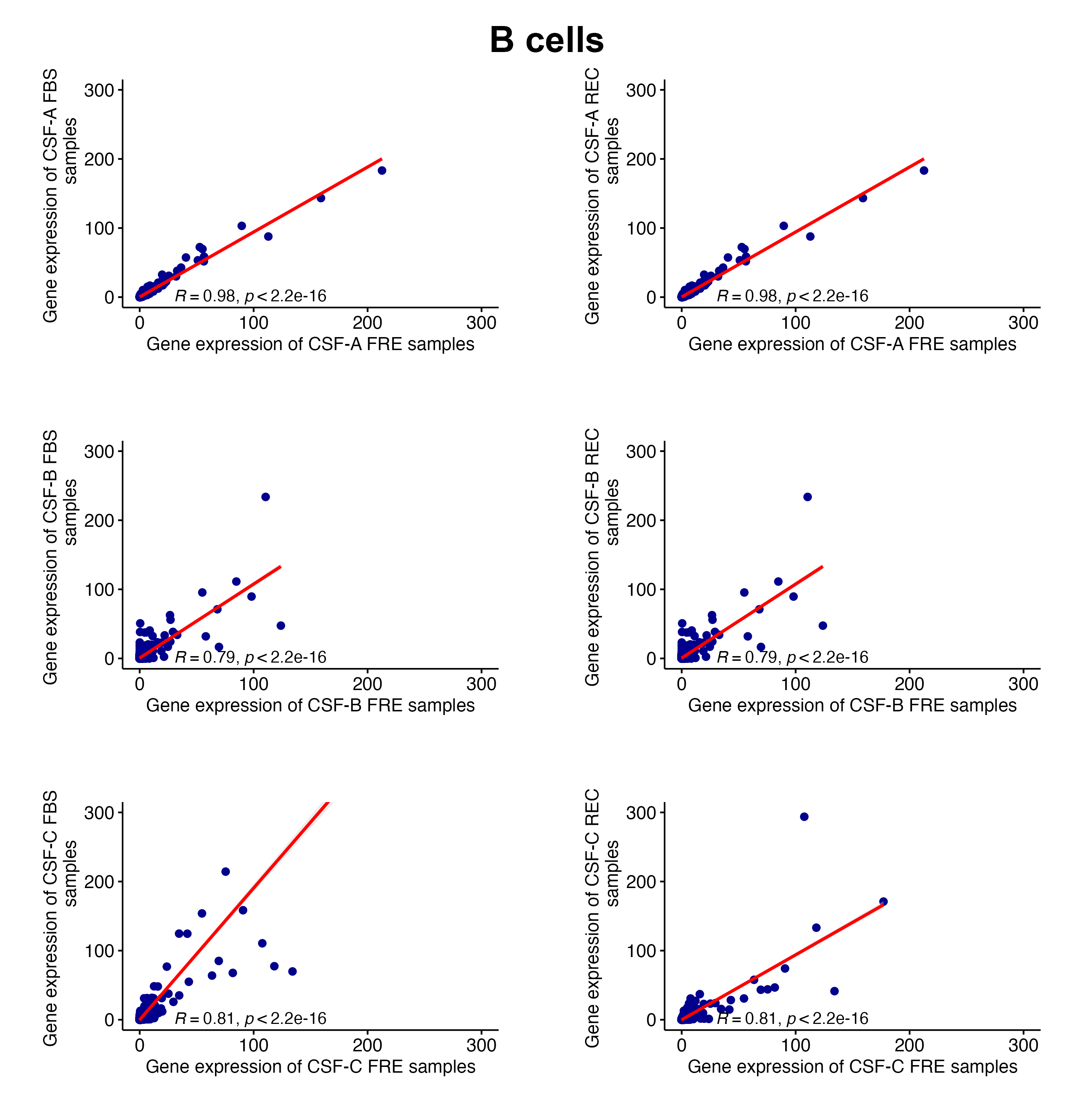

Supplement: Supplementary file 1 — Additional file 1: Fig. S1. Representative images of the CSF cell pellet visible post-centrifugation. See image file. A sample devoid of RBC contamination is seen on the left, and one with substantial RBC contamination is seen on the right. Table S1. Donor demographics and sample information. See Excel file. Subject IDs, Age, Sex, and number of individual reactions tested from FRE, FBS, REC, and DNA samples listed. Table S2. Quality control metrics pre- and post-filtration. See Excel file. QC filtering: Cells having 200 – 4500 umi counts, < 20% mito genes detected were retained. 2000 high variable genes were used for clustering. Columns: Total_cells.before.filtering: cells pre-filtering; cells after filtering: cells remaining after application of above filters; Total_genes: Genes per sample, avg_numi: average number of umi counts before filtering; numi after filtering: average number of umi counts after filtering; avg_mito: average mitochondrial genes in each sample before filtering; mito after filtering: average number of mitochondrial genes in each sample after filtering. Fig. S2. Cellular features, UMI, mitochondrial reads, and cellular stress-related gene expression. See image file. All graphs include data from CSF A, B, and C. A–C Density plot showing the distribution of the number of transcriptional features (genes), number of Unique Molecular Identifiers (UMI), and percentage of overall gene expression attributed to mitochondrial genes, respectively. D–F Violin plots of HSPA1A, HSPA1B, and HSP90AA1 normalized gene expression levels, respectively. Table S3. Cell type designations, proportions and mean predicted scores. See Excel file. Mean predicted score is the Azimuth-derived confidence score for a given annotation. ‘All’ refers to the total of all cell types (L1 or L2). Columns E-L reference L1 designations (used throughout the manuscript), and columns M-AP list L2 designations (not used elsewhere in the manuscript). Summary information for samples by fresh [file 12974_2024_3047_MOESM1_ESM.zip › S9_Fig.jpg]

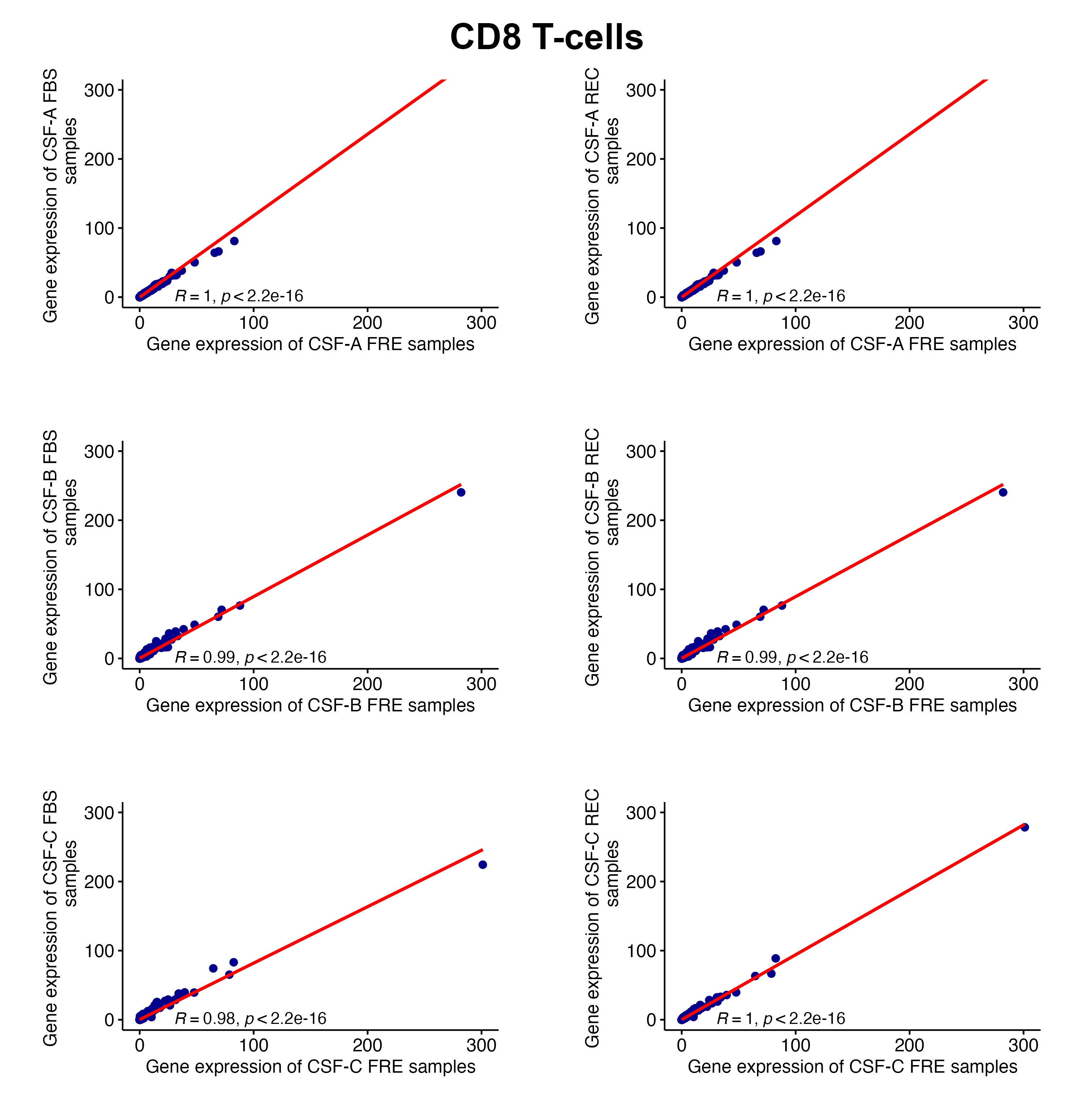

Supplement: Supplementary file 1 — Additional file 1: Fig. S1. Representative images of the CSF cell pellet visible post-centrifugation. See image file. A sample devoid of RBC contamination is seen on the left, and one with substantial RBC contamination is seen on the right. Table S1. Donor demographics and sample information. See Excel file. Subject IDs, Age, Sex, and number of individual reactions tested from FRE, FBS, REC, and DNA samples listed. Table S2. Quality control metrics pre- and post-filtration. See Excel file. QC filtering: Cells having 200 – 4500 umi counts, < 20% mito genes detected were retained. 2000 high variable genes were used for clustering. Columns: Total_cells.before.filtering: cells pre-filtering; cells after filtering: cells remaining after application of above filters; Total_genes: Genes per sample, avg_numi: average number of umi counts before filtering; numi after filtering: average number of umi counts after filtering; avg_mito: average mitochondrial genes in each sample before filtering; mito after filtering: average number of mitochondrial genes in each sample after filtering. Fig. S2. Cellular features, UMI, mitochondrial reads, and cellular stress-related gene expression. See image file. All graphs include data from CSF A, B, and C. A–C Density plot showing the distribution of the number of transcriptional features (genes), number of Unique Molecular Identifiers (UMI), and percentage of overall gene expression attributed to mitochondrial genes, respectively. D–F Violin plots of HSPA1A, HSPA1B, and HSP90AA1 normalized gene expression levels, respectively. Table S3. Cell type designations, proportions and mean predicted scores. See Excel file. Mean predicted score is the Azimuth-derived confidence score for a given annotation. ‘All’ refers to the total of all cell types (L1 or L2). Columns E-L reference L1 designations (used throughout the manuscript), and columns M-AP list L2 designations (not used elsewhere in the manuscript). Summary information for samples by fresh [file 12974_2024_3047_MOESM1_ESM.zip › S8_Fig.jpg]

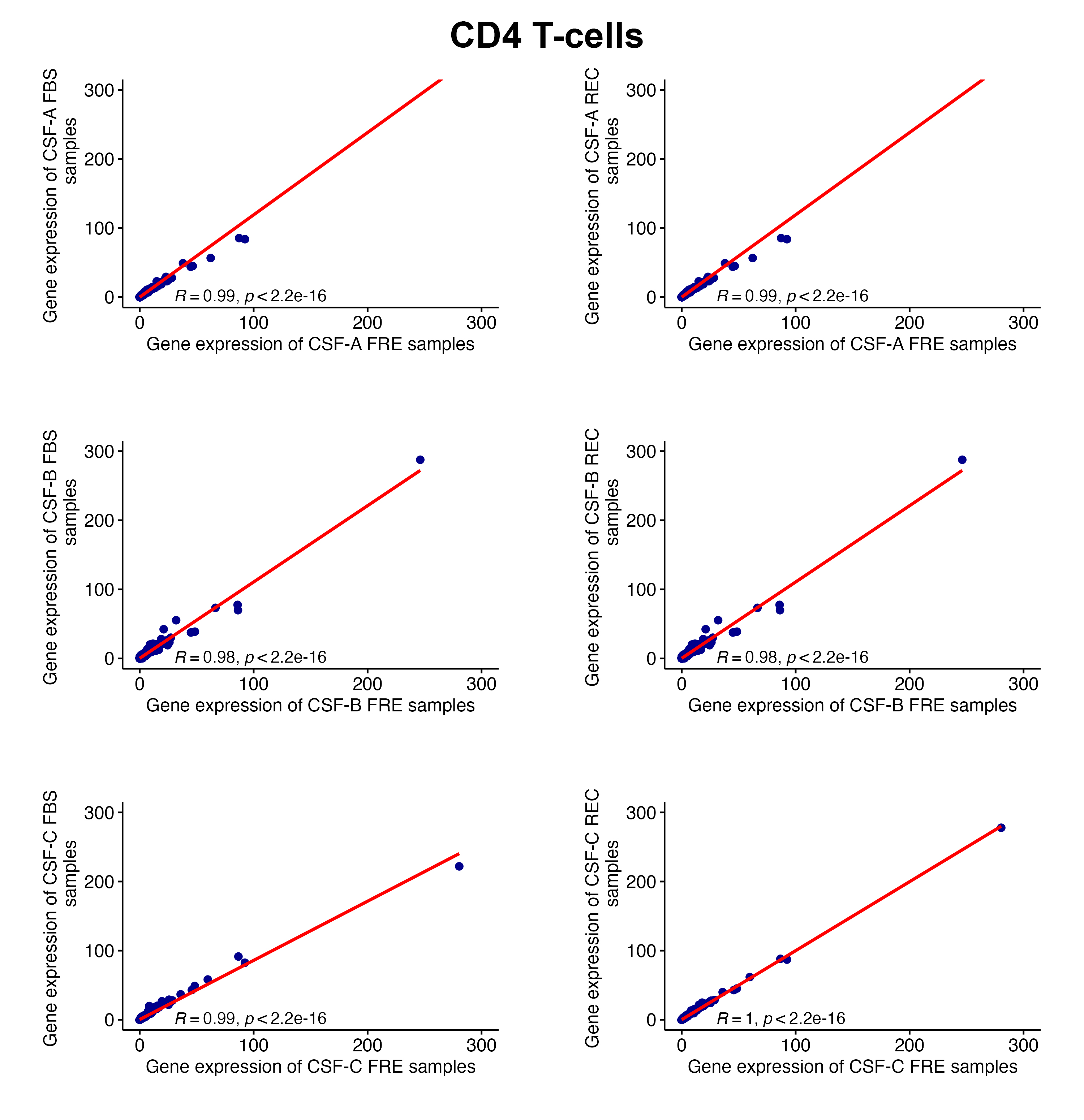

Supplement: Supplementary file 1 — Additional file 1: Fig. S1. Representative images of the CSF cell pellet visible post-centrifugation. See image file. A sample devoid of RBC contamination is seen on the left, and one with substantial RBC contamination is seen on the right. Table S1. Donor demographics and sample information. See Excel file. Subject IDs, Age, Sex, and number of individual reactions tested from FRE, FBS, REC, and DNA samples listed. Table S2. Quality control metrics pre- and post-filtration. See Excel file. QC filtering: Cells having 200 – 4500 umi counts, < 20% mito genes detected were retained. 2000 high variable genes were used for clustering. Columns: Total_cells.before.filtering: cells pre-filtering; cells after filtering: cells remaining after application of above filters; Total_genes: Genes per sample, avg_numi: average number of umi counts before filtering; numi after filtering: average number of umi counts after filtering; avg_mito: average mitochondrial genes in each sample before filtering; mito after filtering: average number of mitochondrial genes in each sample after filtering. Fig. S2. Cellular features, UMI, mitochondrial reads, and cellular stress-related gene expression. See image file. All graphs include data from CSF A, B, and C. A–C Density plot showing the distribution of the number of transcriptional features (genes), number of Unique Molecular Identifiers (UMI), and percentage of overall gene expression attributed to mitochondrial genes, respectively. D–F Violin plots of HSPA1A, HSPA1B, and HSP90AA1 normalized gene expression levels, respectively. Table S3. Cell type designations, proportions and mean predicted scores. See Excel file. Mean predicted score is the Azimuth-derived confidence score for a given annotation. ‘All’ refers to the total of all cell types (L1 or L2). Columns E-L reference L1 designations (used throughout the manuscript), and columns M-AP list L2 designations (not used elsewhere in the manuscript). Summary information for samples by fresh [file 12974_2024_3047_MOESM1_ESM.zip › S7_Fig.jpg]

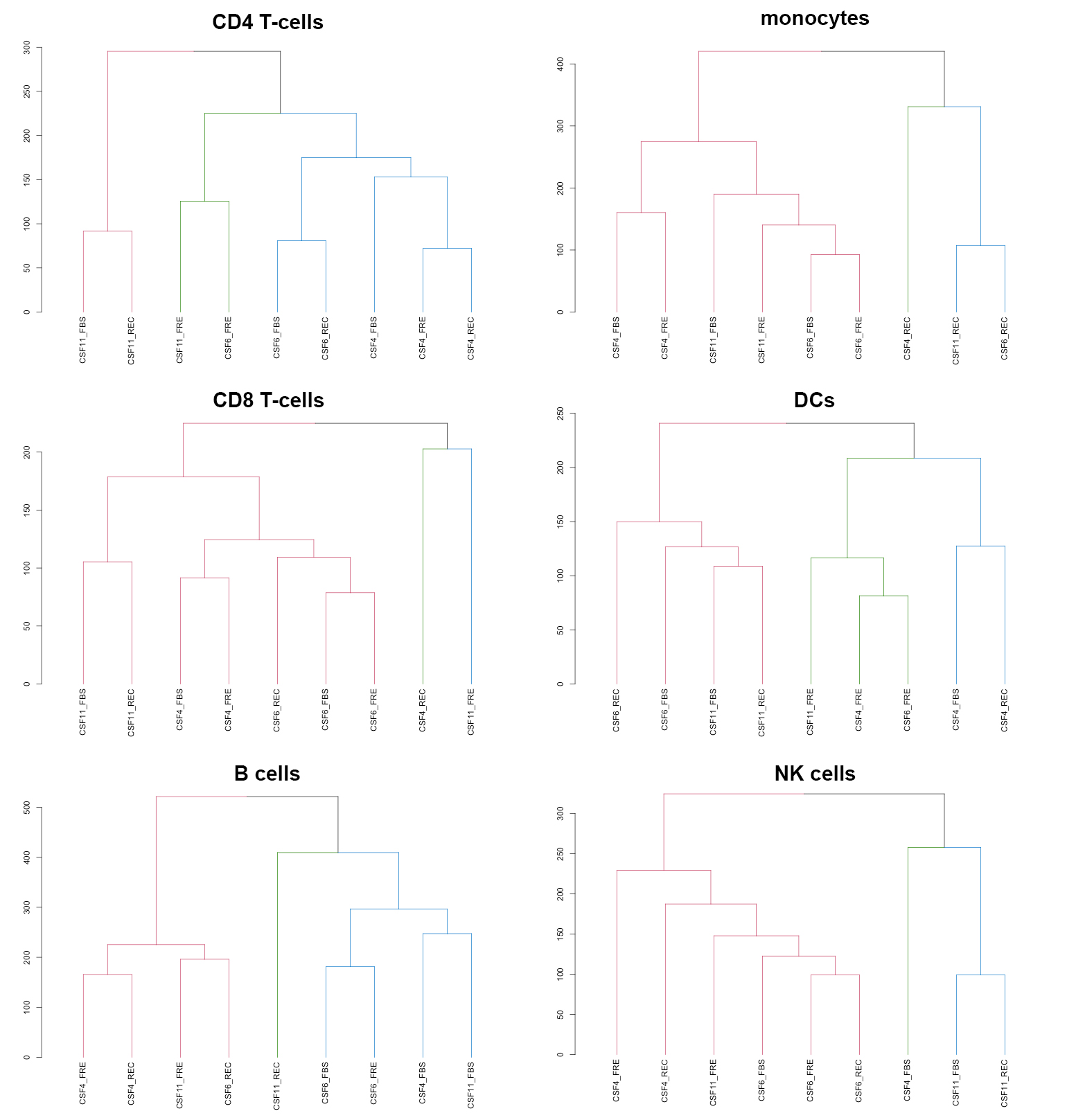

Supplement: Supplementary file 1 — Additional file 1: Fig. S1. Representative images of the CSF cell pellet visible post-centrifugation. See image file. A sample devoid of RBC contamination is seen on the left, and one with substantial RBC contamination is seen on the right. Table S1. Donor demographics and sample information. See Excel file. Subject IDs, Age, Sex, and number of individual reactions tested from FRE, FBS, REC, and DNA samples listed. Table S2. Quality control metrics pre- and post-filtration. See Excel file. QC filtering: Cells having 200 – 4500 umi counts, < 20% mito genes detected were retained. 2000 high variable genes were used for clustering. Columns: Total_cells.before.filtering: cells pre-filtering; cells after filtering: cells remaining after application of above filters; Total_genes: Genes per sample, avg_numi: average number of umi counts before filtering; numi after filtering: average number of umi counts after filtering; avg_mito: average mitochondrial genes in each sample before filtering; mito after filtering: average number of mitochondrial genes in each sample after filtering. Fig. S2. Cellular features, UMI, mitochondrial reads, and cellular stress-related gene expression. See image file. All graphs include data from CSF A, B, and C. A–C Density plot showing the distribution of the number of transcriptional features (genes), number of Unique Molecular Identifiers (UMI), and percentage of overall gene expression attributed to mitochondrial genes, respectively. D–F Violin plots of HSPA1A, HSPA1B, and HSP90AA1 normalized gene expression levels, respectively. Table S3. Cell type designations, proportions and mean predicted scores. See Excel file. Mean predicted score is the Azimuth-derived confidence score for a given annotation. ‘All’ refers to the total of all cell types (L1 or L2). Columns E-L reference L1 designations (used throughout the manuscript), and columns M-AP list L2 designations (not used elsewhere in the manuscript). Summary information for samples by fresh [file 12974_2024_3047_MOESM1_ESM.zip › S6_Fig.jpg]

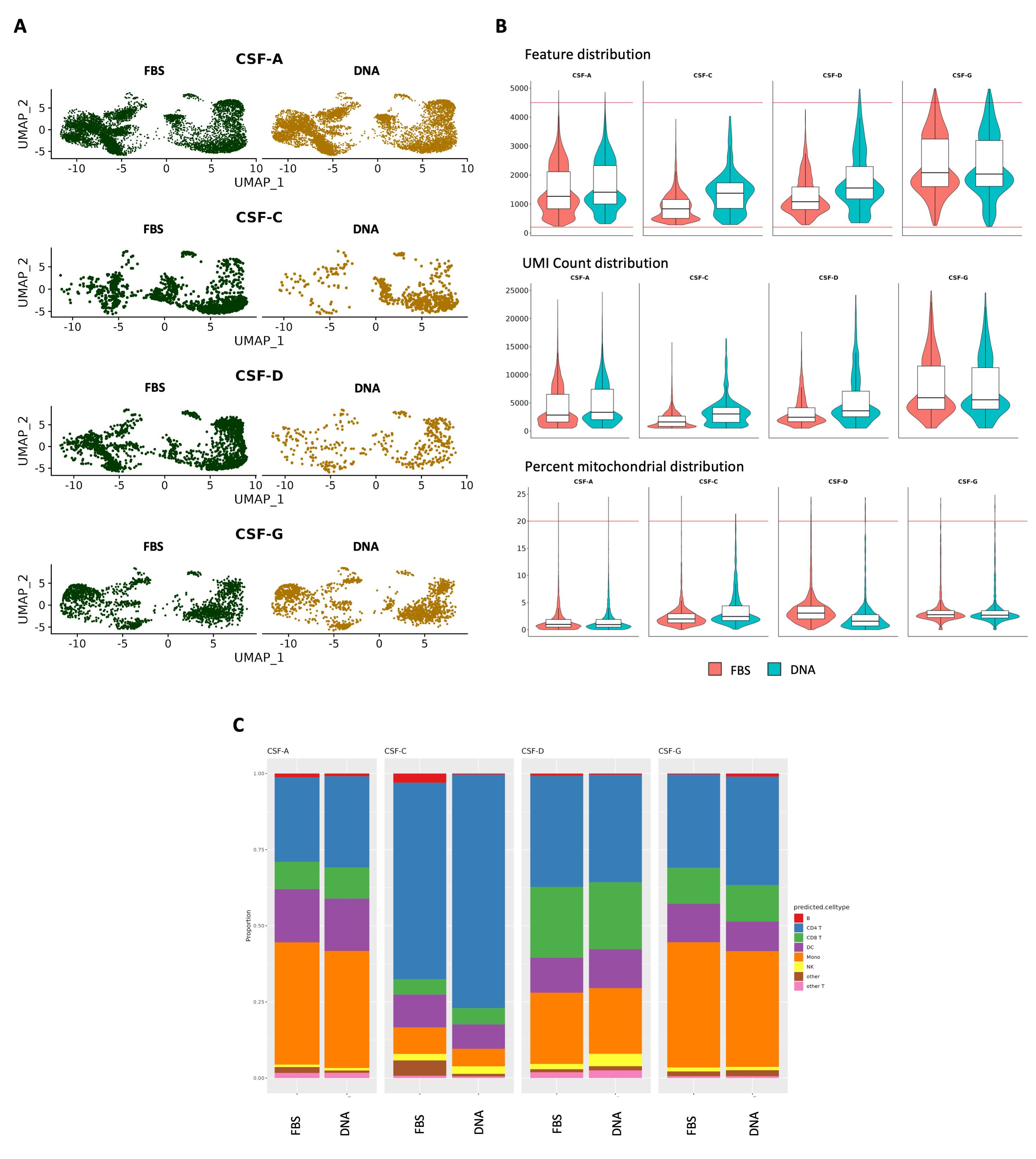

Supplement: Supplementary file 1 — Additional file 1: Fig. S1. Representative images of the CSF cell pellet visible post-centrifugation. See image file. A sample devoid of RBC contamination is seen on the left, and one with substantial RBC contamination is seen on the right. Table S1. Donor demographics and sample information. See Excel file. Subject IDs, Age, Sex, and number of individual reactions tested from FRE, FBS, REC, and DNA samples listed. Table S2. Quality control metrics pre- and post-filtration. See Excel file. QC filtering: Cells having 200 – 4500 umi counts, < 20% mito genes detected were retained. 2000 high variable genes were used for clustering. Columns: Total_cells.before.filtering: cells pre-filtering; cells after filtering: cells remaining after application of above filters; Total_genes: Genes per sample, avg_numi: average number of umi counts before filtering; numi after filtering: average number of umi counts after filtering; avg_mito: average mitochondrial genes in each sample before filtering; mito after filtering: average number of mitochondrial genes in each sample after filtering. Fig. S2. Cellular features, UMI, mitochondrial reads, and cellular stress-related gene expression. See image file. All graphs include data from CSF A, B, and C. A–C Density plot showing the distribution of the number of transcriptional features (genes), number of Unique Molecular Identifiers (UMI), and percentage of overall gene expression attributed to mitochondrial genes, respectively. D–F Violin plots of HSPA1A, HSPA1B, and HSP90AA1 normalized gene expression levels, respectively. Table S3. Cell type designations, proportions and mean predicted scores. See Excel file. Mean predicted score is the Azimuth-derived confidence score for a given annotation. ‘All’ refers to the total of all cell types (L1 or L2). Columns E-L reference L1 designations (used throughout the manuscript), and columns M-AP list L2 designations (not used elsewhere in the manuscript). Summary information for samples by fresh [file 12974_2024_3047_MOESM1_ESM.zip › S5_Fig.jpg]

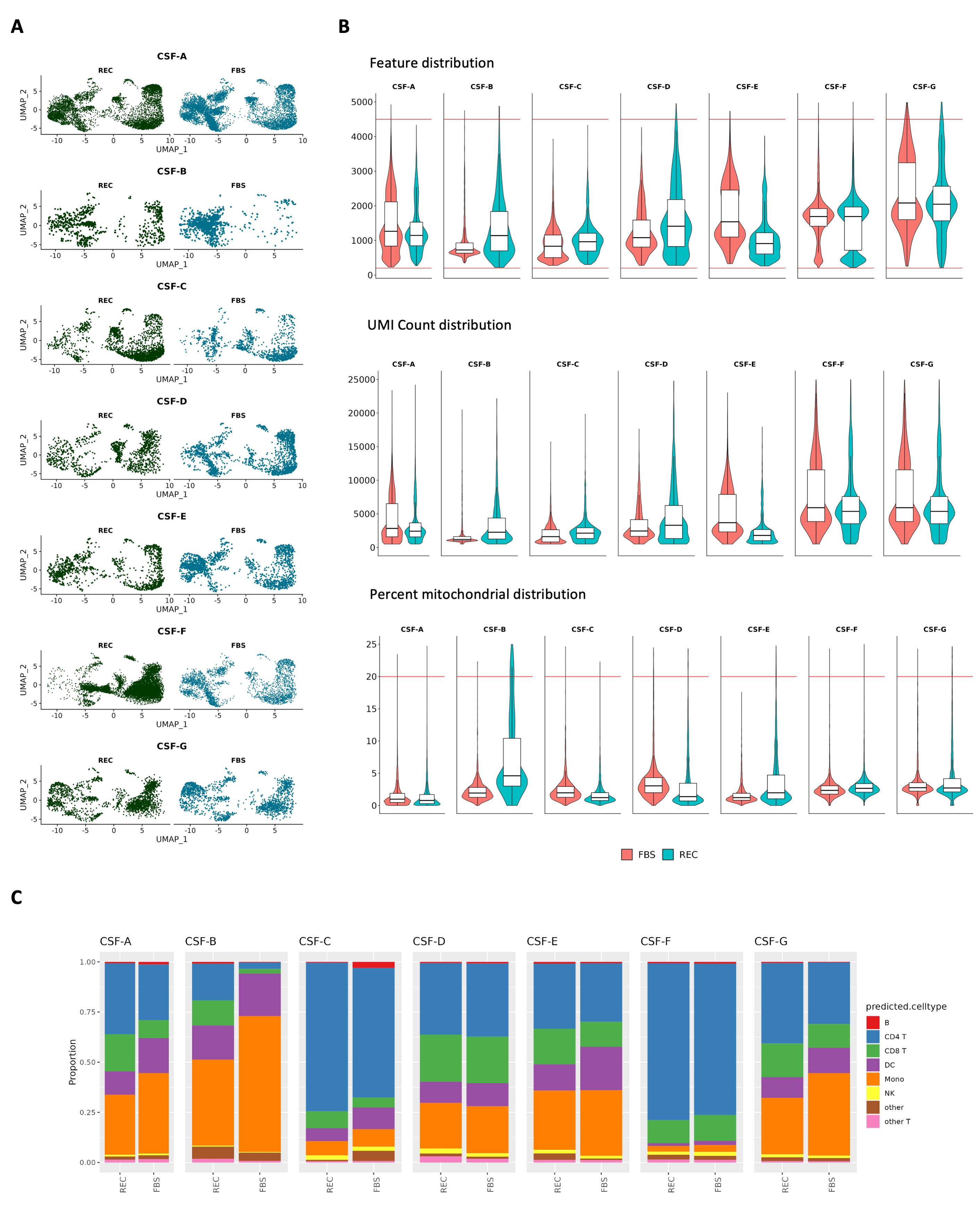

Supplement: Supplementary file 1 — Additional file 1: Fig. S1. Representative images of the CSF cell pellet visible post-centrifugation. See image file. A sample devoid of RBC contamination is seen on the left, and one with substantial RBC contamination is seen on the right. Table S1. Donor demographics and sample information. See Excel file. Subject IDs, Age, Sex, and number of individual reactions tested from FRE, FBS, REC, and DNA samples listed. Table S2. Quality control metrics pre- and post-filtration. See Excel file. QC filtering: Cells having 200 – 4500 umi counts, < 20% mito genes detected were retained. 2000 high variable genes were used for clustering. Columns: Total_cells.before.filtering: cells pre-filtering; cells after filtering: cells remaining after application of above filters; Total_genes: Genes per sample, avg_numi: average number of umi counts before filtering; numi after filtering: average number of umi counts after filtering; avg_mito: average mitochondrial genes in each sample before filtering; mito after filtering: average number of mitochondrial genes in each sample after filtering. Fig. S2. Cellular features, UMI, mitochondrial reads, and cellular stress-related gene expression. See image file. All graphs include data from CSF A, B, and C. A–C Density plot showing the distribution of the number of transcriptional features (genes), number of Unique Molecular Identifiers (UMI), and percentage of overall gene expression attributed to mitochondrial genes, respectively. D–F Violin plots of HSPA1A, HSPA1B, and HSP90AA1 normalized gene expression levels, respectively. Table S3. Cell type designations, proportions and mean predicted scores. See Excel file. Mean predicted score is the Azimuth-derived confidence score for a given annotation. ‘All’ refers to the total of all cell types (L1 or L2). Columns E-L reference L1 designations (used throughout the manuscript), and columns M-AP list L2 designations (not used elsewhere in the manuscript). Summary information for samples by fresh [file 12974_2024_3047_MOESM1_ESM.zip › S4_Fig.jpg]

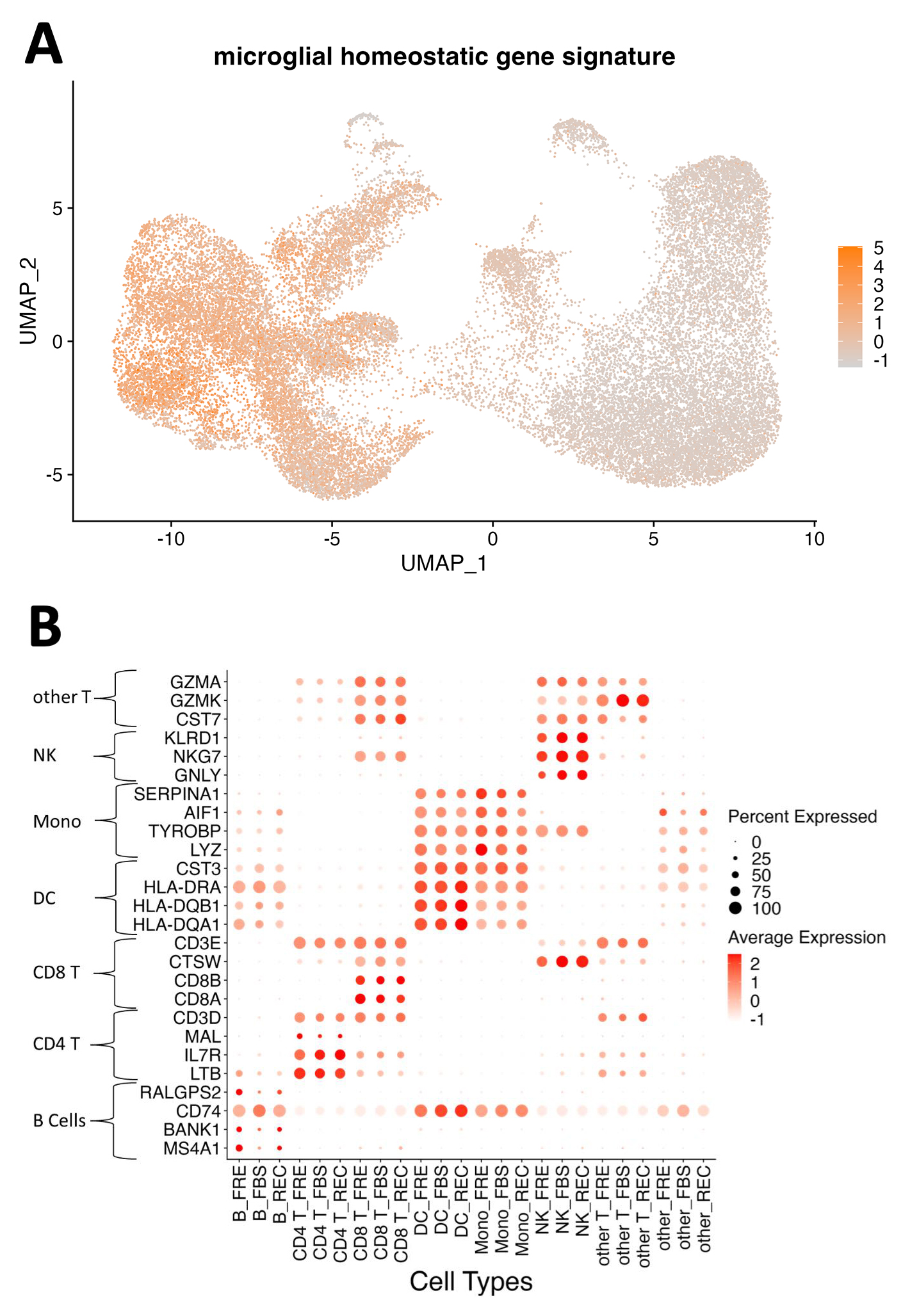

Supplement: Supplementary file 1 — Additional file 1: Fig. S1. Representative images of the CSF cell pellet visible post-centrifugation. See image file. A sample devoid of RBC contamination is seen on the left, and one with substantial RBC contamination is seen on the right. Table S1. Donor demographics and sample information. See Excel file. Subject IDs, Age, Sex, and number of individual reactions tested from FRE, FBS, REC, and DNA samples listed. Table S2. Quality control metrics pre- and post-filtration. See Excel file. QC filtering: Cells having 200 – 4500 umi counts, < 20% mito genes detected were retained. 2000 high variable genes were used for clustering. Columns: Total_cells.before.filtering: cells pre-filtering; cells after filtering: cells remaining after application of above filters; Total_genes: Genes per sample, avg_numi: average number of umi counts before filtering; numi after filtering: average number of umi counts after filtering; avg_mito: average mitochondrial genes in each sample before filtering; mito after filtering: average number of mitochondrial genes in each sample after filtering. Fig. S2. Cellular features, UMI, mitochondrial reads, and cellular stress-related gene expression. See image file. All graphs include data from CSF A, B, and C. A–C Density plot showing the distribution of the number of transcriptional features (genes), number of Unique Molecular Identifiers (UMI), and percentage of overall gene expression attributed to mitochondrial genes, respectively. D–F Violin plots of HSPA1A, HSPA1B, and HSP90AA1 normalized gene expression levels, respectively. Table S3. Cell type designations, proportions and mean predicted scores. See Excel file. Mean predicted score is the Azimuth-derived confidence score for a given annotation. ‘All’ refers to the total of all cell types (L1 or L2). Columns E-L reference L1 designations (used throughout the manuscript), and columns M-AP list L2 designations (not used elsewhere in the manuscript). Summary information for samples by fresh [file 12974_2024_3047_MOESM1_ESM.zip › S3_Fig.jpg]

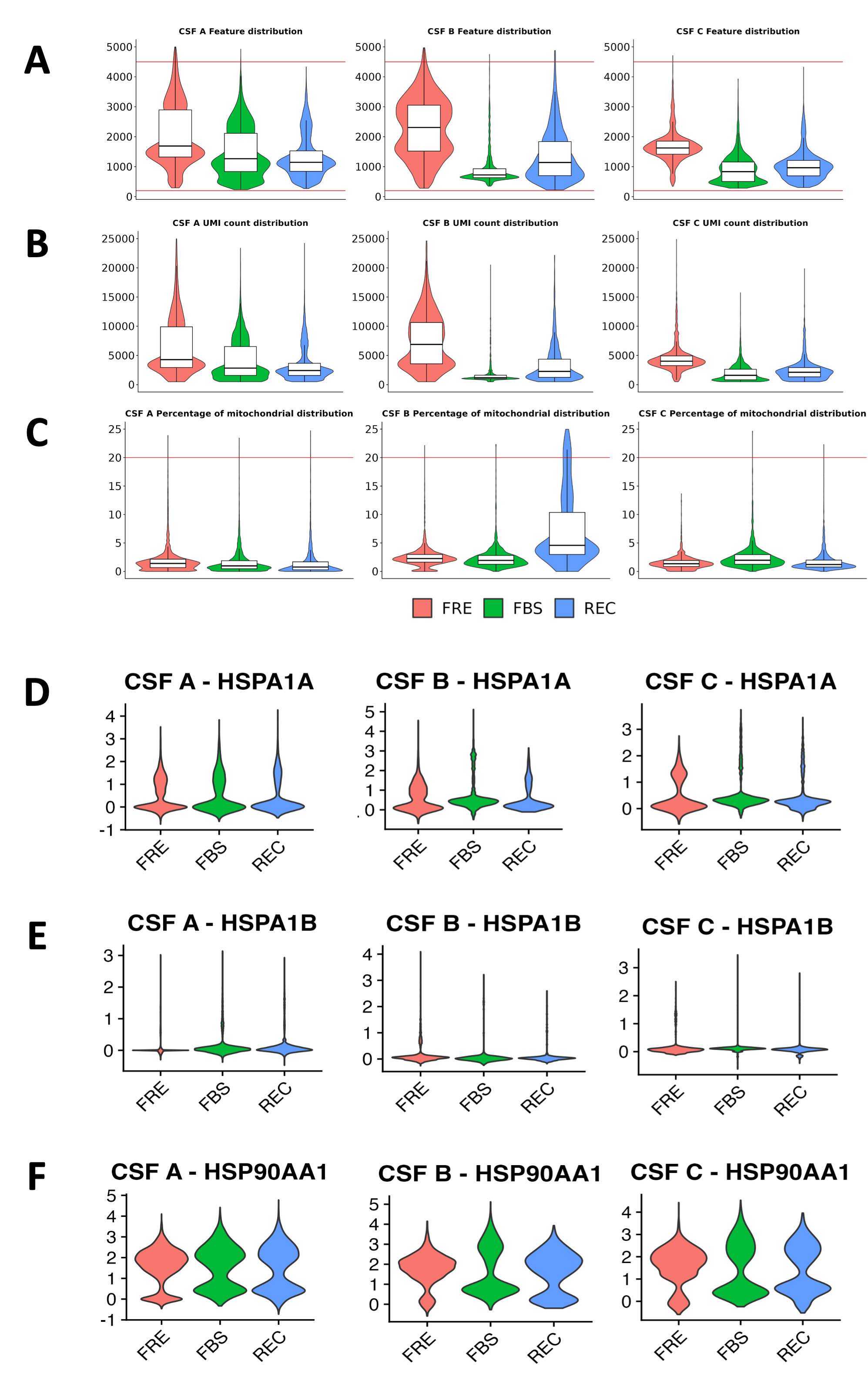

Supplement: Supplementary file 1 — Additional file 1: Fig. S1. Representative images of the CSF cell pellet visible post-centrifugation. See image file. A sample devoid of RBC contamination is seen on the left, and one with substantial RBC contamination is seen on the right. Table S1. Donor demographics and sample information. See Excel file. Subject IDs, Age, Sex, and number of individual reactions tested from FRE, FBS, REC, and DNA samples listed. Table S2. Quality control metrics pre- and post-filtration. See Excel file. QC filtering: Cells having 200 – 4500 umi counts, < 20% mito genes detected were retained. 2000 high variable genes were used for clustering. Columns: Total_cells.before.filtering: cells pre-filtering; cells after filtering: cells remaining after application of above filters; Total_genes: Genes per sample, avg_numi: average number of umi counts before filtering; numi after filtering: average number of umi counts after filtering; avg_mito: average mitochondrial genes in each sample before filtering; mito after filtering: average number of mitochondrial genes in each sample after filtering. Fig. S2. Cellular features, UMI, mitochondrial reads, and cellular stress-related gene expression. See image file. All graphs include data from CSF A, B, and C. A–C Density plot showing the distribution of the number of transcriptional features (genes), number of Unique Molecular Identifiers (UMI), and percentage of overall gene expression attributed to mitochondrial genes, respectively. D–F Violin plots of HSPA1A, HSPA1B, and HSP90AA1 normalized gene expression levels, respectively. Table S3. Cell type designations, proportions and mean predicted scores. See Excel file. Mean predicted score is the Azimuth-derived confidence score for a given annotation. ‘All’ refers to the total of all cell types (L1 or L2). Columns E-L reference L1 designations (used throughout the manuscript), and columns M-AP list L2 designations (not used elsewhere in the manuscript). Summary information for samples by fresh [file 12974_2024_3047_MOESM1_ESM.zip › S2_Fig.jpg]
